# Targeting epigenetic pathways in acute myeloid leukemia and myelodysplastic syndrome: a systematic review of hypomethylating agents trials

**DOI:** 10.1186/s13148-016-0233-2

**Published:** 2016-06-14

**Authors:** Seongseok Yun, Nicole D. Vincelette, Ivo Abraham, Keith D. Robertson, Martin E. Fernandez-Zapico, Mrinal M. Patnaik

**Affiliations:** Department of Medicine, University of Arizona, 1501 N. Campbell Ave., Tucson, AZ 85721 USA; Hematology and Oncology, H. Lee Moffitt Cancer Center, Tampa, FL 12902 USA; Molecular Pharmacology and Experimental Therapeutics, Department of Medicine, Mayo Clinic, Rochester, MN 55905 USA; Center for Health Outcomes and PharmacoEconomic Research, University of Arizona, Tucson, AZ 85721 USA; Arizona Cancer Center, University of Arizona, Tucson, AZ 85721 USA; Pharmacology, Department of Medicine, Mayo Clinic, Rochester, MN 55905 USA; Schulze Center for Novel Therapeutics, Department of Medicine, Mayo Clinic, Rochester, MN 55905 USA; Division of Hematology, Department of Medicine, Mayo Clinic, Rochester, MN 55905 USA

**Keywords:** AML, MDS, DNA hypomethylating agents, Epigenetics

## Abstract

**Background:**

Aberrant DNA methylation has been identified as a key molecular event regulating the pathogenesis of myelodysplastic syndromes (MDS); myeloid neoplasms with an inherent risk of transformation to acute myeloid leukemia (AML). Based on the above findings, DNA hypomethylating agents (HMA) have been widely used to treat AML and MDS, especially in elderly patients and in those who are not eligible for allogeneic stem cell transplantation (SCT). Our goal was to determine if there is any therapeutic advantage of HMA vs. conventional care regimens (CCR) and indirectly compare the efficacy of azacitidine and decitabine in this patient population.

**Methods:**

Eligible studies were limited to randomized controlled trials comparing HMA to CCR in adult patients with AML or MDS.

**Results:**

Overall survival (OS) rate was 33.2 vs. 21.4 % (RR 0.83, 95 % CI 0.71–0.98) and overall response rate (ORR) 23.7 vs. 13.4 % (RR 0.87, 95 % CI 0.81–0.93) for HMA and CCR, respectively. In subgroup analyses, only azacitidine treatment showed OS improvement (RR 0.75, 95 % CI 0.64–0.98) and not decitabine. Cytogenetic risk or bone marrow blast count did not have independent prognostic impact.

**Conclusion:**

Collectively, these results demonstrate that HMA have superior outcomes compared to CCR and suggest that azacitidine in comparison to decitabine, may be more effective.

**Electronic supplementary material:**

The online version of this article (doi:10.1186/s13148-016-0233-2) contains supplementary material, which is available to authorized users.

## Background

Myelodysplastic syndromes (MDS) are clonal hematopoietic stem cell disorders characterized by peripheral blood cytopenias, hypercellular bone marrows, and an inherent predisposition to transform to acute myeloid leukemia (AML) [1]. MDS are commonly associated with aging (age-related acquisition of genomic and epigenetic changes), environmental carcinogens, chemotherapy, and radiation exposure (therapy-related MDS) [2]. AML, an aggressive stem cell malignancy, with an annual incidence of 18,860 cases in the USA in 2014 [3], is characterized by ≥20 % bone marrow (BM) blasts and very poor outcomes with chemotherapy [4]. Although allogeneic stem cell transplantation (SCT) is the only curative treatment for high risk MDS and AML [5, 6], many patients are not eligible for transplantation due to advanced age, associated co-morbidities, and a limited donor pool [7]. Thus, there is an urgent need to develop new therapeutic approaches for these patients.

In the last decade, attention has turned to epigenetic changes in MDS/AML, especially aberrant DNA methylation, a molecular process playing a role in the regulation and expression of tumor suppressor genes and oncogenes, promoting dysplasia and blast transformation [8, 9]. These epigenetic modifications are distinguished from genetic mutations by their reversibility, making them potential therapeutic targets. Accordingly, the clinical and biological efficacy of hypomethylating agents (HMA) have been demonstrated in several in vitro/in vivo studies and clinical trials [8, 10–13]. Despite the promising initial treatment responses, the survival outcome data with HMA have been inconsistent. Here, we provide a systematic review and pooled analysis of randomized clinical trials (RCT) comparing the outcomes of HMA vs. conventional care regimens (CCR) in patients with AML or MDS. CCR include best supportive care (BSC), intensive chemotherapy (IC), and low dose cytarabine (LDAC).

## Materials and methods

### Study selection criteria

Eligible studies were (1) RCTs, (2) assessing adult patients age ≥18 years with (3) morphologically proven diagnosis of AML or MDS with no previous allogeneic SCT, (4) treated with either HMA (azacitidine or decitabine) or CCR (BSC, LDAC or IC) in a setting of first-line treatment, and (5) including OS and treatment response outcomes. Trials were used only once in the analysis using the most updated available data.

### Data sources

Literature search and review of relevant articles were limited to human studies. Key words included AML, MDS, azacitidine, and decitabine (Additional file 1: Table S1). Relevant studies were identified by searching PubMed, EMBASE, and Cochrane Database of Systematic Reviews up to October 2015. Additional relevant abstracts from the American Society of Hematology, the American Society of Clinical Oncology, and the European Hematology Association were also included into the literature search. A bibliography of identified articles and additional literatures from relevant references were further investigated manually to identify any relevant trials.

### Data extraction

Two reviewers (SY and NDV) independently extracted data with a piloted extraction form. Any disagreement was resolved by consensus with other co-authors after review of full text.

### Data items

The following information was extracted from individual trial reports: publication year, inclusion/exclusion criteria, sample size, median age, French-American-British (FAB) and World Health Organization (WHO) classification, BM blast count, cytogenetic risk categories, supportive care regimens, median follow-up, OS, treatment response, and mortality attributed to disease progression. Extracted from each study report were the number of patients treated with HMA or CCR, the proportion of patients with events (death, complete remission (CR), partial remission (PR)), subgroup data, hazard ratio (HR), 95 % CI, and *p* values. The primary outcome in this analysis was OS rate, and the secondary outcome was ORR (defined as rate of CR or PR). Trials reported outcomes with variable follow-up of 1–2 years; however, data from all studies were analyzed together with an assumption that median survival of AML patients without intensive chemotherapy or allogeneic SCT is less than 2 years in high-risk AML and MDS patients.

### Assessment of bias risk

We used the Cochrane Collaboration’s tool [14], which evaluates random sequence generation, allocation concealment, blinding, incomplete outcome data, selective reporting, and other source of bias.

### Statistical analysis

Statistical analyses were performed as described in a previous meta-analysis [15]. Briefly, the Cochrane Q statistic was used to estimate statistical heterogeneity, and the *I*^2^ statistic was used to quantify inconsistency. The assumption of homogeneity was considered invalid if *p* < 0.10 and treatment effects were calculated with a random effects model. The funnel plot method was applied to assess publication bias. A two-sided *p* ≤ 0.05 was considered statistically significant in the RR and HR analysis without multiplicity correction. Pre-defined criteria including experimental agents (azacitidine vs. decitabine), cytogenetic risk, and BM blast count were used for the subgroup analyses to explore heterogeneity and to identify subgroups with differential benefit from HMA treatment (Table 2). RR and HR differences between subgroups were evaluated by regression models. Analysis calculations were performed using RevMan Version 5.3.

## Results

### Search results

**Fig. 1:**
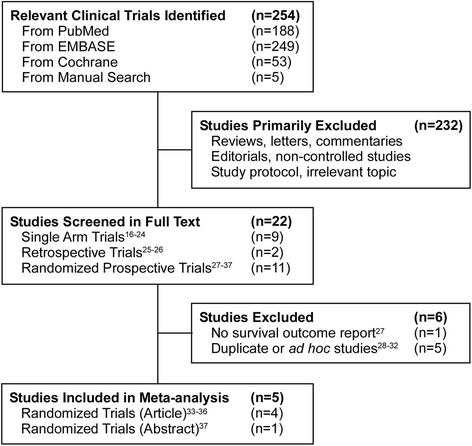
Trial Selection Process for the Systematic Review

Our initial literature search yielded a total 254 potential abstracts (Fig. 1 and Additional file 1: Figure S1). Of this, 232 studies were excluded for being irrelevant to our analysis including editorials, study protocols, and commentaries. Total 22 articles were reviews in full text for their eligibility. Additional nine single arm studies [16–24] and two retrospective studies [25, 26] were excluded from the analysis, as was one study [27] with no survival outcome report and five duplicate or ad hoc studies [28–32]. With careful review of eligibility, a total of five open label multicenter phase III RCTs (four published articles [33–36] and one abstract [37]) were selected for the current analysis. The characteristics of these trials are summarized in Table 1 and Additional file 1: Table S2.

**Table 1:** Characteristics of trials included in the analysis

Study	HMA	Median Age (Range)	FAB classification^a^ (%)	BM Blast (%)	Oligo-blastic AML^b^ (%)	WHO AML (%)	*De novo* AML^c^ (%)	Secondary AML ^c^ (%)	Type of CCR (%)	No. of Patients		Median F/U (mo)	Formula of Experimental Drug
										HMA	CCR		
										Total	Total		
Silverman 2002 [25, 30]	Azacitidine	68 (31-92)	RA: 37 (19) RARS: 8 (4) RAEB: 66 (35) RAEB-T: 45 (24) CMMoL: 14 (7) Others^d^ : 21 (11)	< 30%: 170 (89) ≥ 30%: 19 (10)	45 (24)	67 (35)	0 (0)	67 (35)	BSC: 92 (100)	99	92	NR	Azacitidine: 75 mg/m^2^/d subcutaneous injection in 7 day cycles beginning on days 1, 29, 57, and 85.
Fenaux 2009 [31, 57]	Azacitidine	70 (38-88)	RA: 0 (0) RARS: 0 (0) RAEB: 207 (35) RAEB-T: 123 (24) CMMoL: 11 (7)	< 30%: 356 (99) ≥ 30%: 2 (1)	123 (34)	113 (32)	0 (0)	113 (32)	BSC: 105 (59) LDAC: 49 (27) IC: 25 (14)	179	179	21.1	Azacitidine: 75 mg/m^2^/d subcutaneous injection every 28 days for at least 6 cycles. LDAC: 20 mg/m^2^/d subcutaneous injection for 14 d, every 28 days, at least 4 cycles. IC: cytarabine 100-200 mg/m^2^/d continuous intravenous infusion for 7 days plus 3 days of either intravenous daunorubicin 40-65 mg/m^2^/d or idarubicin 9-12 mg/m^2^/d or mitoxantrone 8-12 mg/m^2^/d.
Lubbert 2011 [27, 28]	Decitabine	70 (60-90)	RA: 13 (6) RARS: 5 (2) RAEB: 125 (54) RAEB-T: 75 (32) CMMoL: 14 (6) AML: 2 (1)	< 30%: 232 (99) ≥ 30%: 2 (1)	75 (32)	77 (32)	0 (0)	77 (32)	BSC: 114 (100)	119	114	30	Decitabine: 15 mg/m^2^ in 2 doses, intravenous infusion every 8h for 3 d. This treatment cycle was repeated every 6 wks.
Kantarjian 2012 [29]	Decitabine	73 (64-91)	RA: 0 (0) RARS: 0 (0) RAEB: 0 (0) RAEB-T: 123 (25) AML: 363 (75) CMMoL: 0 (0)	< 30%: 123 (25) ≥ 30%: 347 (72)	123 (25)	485 (100)	312 (64)	173 (36)	BSC: 28 (12) LDAC: 215 (88)	242	243	NR	Decitabine: 20 mg/m^2^/d intravenous infusion for 5 d. This treatment cycle was repeated every 4 wks. LDAC: 20 mg/m^2^/d subcutaneous injection for 10 consecutive days every 4 wks.
Dombret 2014 [26, 32]	Azacitidine	75 (NR)	RA: 0 (0) RARS: 0 (0) RAEB: 0 (0) RAEB-T: 0 (0) AML: 488 (100)	< 30%: 0 (0) ≥ 30%: 488 (100)	0 (0)	488 (100)	NR ^e^	NR ^e^	BSC: 45 (18) LDAC: 158 (64) IC: 44 (18)	241	247	NR	Azacitidine: 75 mg/m^2^/d subcutaneous for 7 d, 28 d cycle. LDAC: 20 mg/m^2^ subcutaneous injection twice a day for 10 days with every 28 days cycle. IC: standard 7 + 3 regimen

^a^ FAB classification: RA (refractory anemia), RARS (refractory anemia with ring sideroblasts), RAEB (refractory anemia with excessive blasts), RAEB-t (RAEB in transformation with BM blast 21-30%)), CMMoL (chronic myelomonocytic leukemia), AML (acute myeloid leukemia with BM blast more thatn 30%)

^b^ Oligoblasatic AML: BM blast counts 20-30%

^c^ *De novo* AML: the definition of *de novo* and secondary AML followed the revised recommendations of the International Working Group [58] (no clinical history of MDS, MPD or exposure to potential leukemogenic treatment or agents) and followed WHO classification.

^d^ Others: AML (n = 19), undefined leukemia (n = 1), undefined MDS (n = 1)

^e^ This study included both *de novo* and secondary AML. Total 158 patients had AML with myelodysplastic related change (AML-MRC).

Abbreviation: *NR* (not reported), *HMA* (hypomethlating agents (DNA methyl-transferase inhibitor)), *BSC* (best supportive care), *LDAC* (low dose cytarabine), *IC* (intensive chemotherapy)

### Patients

All trials included patients with morphologically confirmed AML or MDS and age of 18 years or greater. A total of 1755 patients were included in the analysis. Of these, 880 were treated with either azacitidine (*n* = 519) or decitabine (*n* = 361) and 875 with CCR including BSC (*n* = 384), LDAC (*n* = 422), and IC (*n* = 69) (Table 1). The range of median ages of patients on the selected trials was 68–75 years. Cytogenetic risk stratification was performed following South West Oncology Group (SWOG) [38] and International Prognostic Scoring System (IPSS) [39] categorization of AML and MDS, respectively. The number of patients with BM blast ≥30 %, oligoblastic AML (BM blasts 20–30 %), *de novo* AML, and intermediate/poor risk cytogenetic AML were 858, 366, 312, and 1278, respectively (Additional file 1: Table S2). The numbers of MDS patients with low and intermediate/high IPSS risk categorization were 7 and 636, respectively. Cytogenetic analysis was done on only 42 % of patients in one study [35], while additional studies [35, 37] failed to report subgroup outcomes according to cytogenetic risk and BM blast count. Three studies [33, 35, 36] included both AML and MDS patients and two studies [34, 37] included AML only (by FAB classification). Two trials [34, 37] included *de novo* AML patients without separate outcome report between transformed/secondary vs. *de novo* AML, and one of these studies included 158 patients with AML with myelodysplastic-related change (AML-MRC) [37]. Median follow-up was reported in only two studies [33, 36]. Forty-nine (53 %) CCR patients in one study [35] crossed over to the azacitidine arm, while the remaining studies did not have crossover options.

### RR of OS rate and ORR

**Fig. 2:**
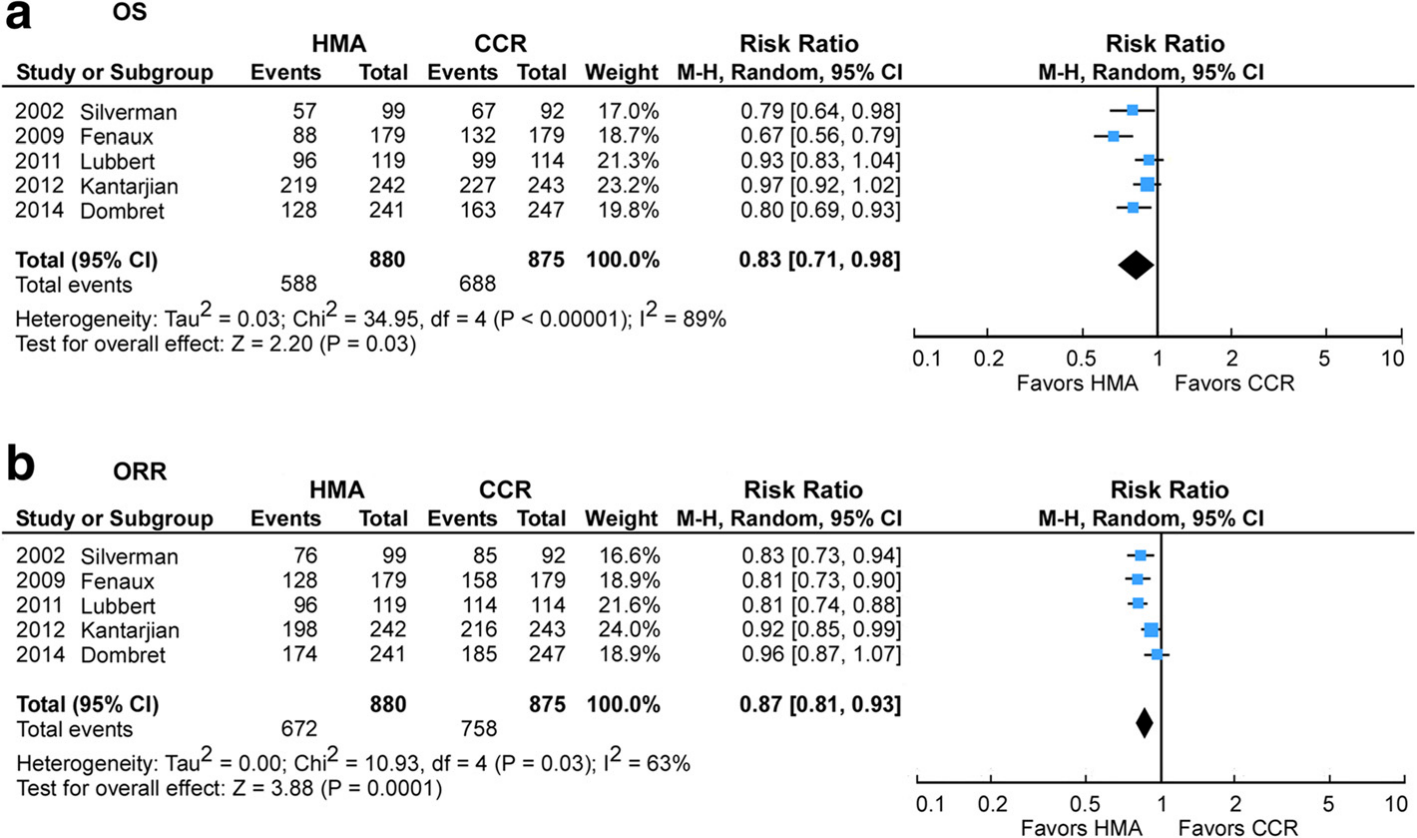
**a** Risk Ratio of the OS Rate. **b** Risk Ratio of the Overall Response Rate

The combined estimate demonstrated an association of HMA treatment with significantly better OS rate of 33.2 vs. 21.4 % (RR 0.83, 95 % CI 0.71–0.98, *p* = 0.03) and higher ORR of 23.7 vs. 13.4 % (RR 0.87, 95 % CI 0.81–0.93, *p* = 0.0001) (Fig. 2). There was significant heterogeneity in OS (*I*^2^ = 89 %, *p* < 0.00001) and ORR (*I*^2^ = 63 %, *p* = 0.03) analyses across studies.

### Subgroup analyses

Azacitidine treatment was associated with significantly better OS compared to CCR (HR 0.67, 95 % CI 0.56–0.79, *p* < 0.00001), while no statistically significant OS benefit was observed in the decitabine treatment group (HR 0.86, 95 % CI 0.73–1.02, *p* = 0.08) (Table 2), partially explaining the heterogeneity in the OS analysis (Additional file 1: Figure S2A). Both azacitidine (RR 0.87, 95 % CI 0.78–0.97, *p* = 0.01) and decitabine (RR 0.86, 95 % CI 0.76–0.98, *p* = 0.03) treatments showed a higher ORR when each was compared to CCR with no RR difference between both treatments relative to CCR (*p* = 0.97) (Additional file 1: Figure S3). There was no statistically significant association of OS with cytogenetic risk, BM blast count, and use of LDAC or IC supplemental to BSC (Table 2 and Additional file 1: Figure S2B-D). Additional subgroup analyses directly comparing the OS rates of HMA and LDAC in AML patients revealed no significant difference (21.8 vs. 12.1 %, RR 0.77, 95 % CI 0.52–1.16, *p* = 0.21) between HMA and LDAC. Azacitidine treatment was associated with significantly better OS rates compared to LDAC (RR 0.66, 95 % CI 0.49–0.87, *p* = 0.004); however, no significant OS benefit over LDAC was seen in the decitabine subgroup (RR 0.97, 95 % CI 0.92–1.02, *p* = 0.24) (Additional file 1: Figure S4 and Table S4).

**Table 2:** Subgroup analysis of overall survival from available data

Subgroup		No. of studies	OS HR (95 % CI)^d^	Weight (%)	Heterogeneity within subgroup	
Criteria	Characteristics				*I* ^2^ (%)	*p* value
Experimental drug	Azacitidine^a^	3	0.67 (0.56, 0.79)	48.3	0	0.38
	Decitabine	2	0.86 (0.73, 1.02)	51.7	0	0.85
	Subgroup difference	*p* = 0.04*				
Cytogenetics risk^b^	Poor risk	3	0.75 (0.56, 1.00)	43.9	26	0.26
	Intermediate risk	3	0.78 (0.40, 1.52)	37.0	65	0.06
	Good risk	2	0.63 (0.42, 0.93)	19.0	0	0.64
	Subgroup difference	*p* = 0.75				
BM blast count^c^	More than 30 %	2	0.79 (0.68, 0.92)	48.9	0	0.32
	Less than 30 %	3	0.82 (0.57, 1.18)	51.1	74	0.02
	Subgroup difference	*p* = 0.85				
Conventional care regimens	BSC only	2	0.76 (0.53, 1.10)	28.4	44	0.18
	BSC and CTx^e^	3	0.73 (0.60, 0.90)	71.6	53	0.12
	Subgroup difference	*p* = 0.85				

^a^One study [25, 30] included only RAEB and RAEB-T for the HR analysis of OS

^b^Two studies [30, 32] did not report subgroup survival outcome data according to cytogenetic risk

^c^One study [30] did not report subgroup survival outcome data according to BM blast count (≥30 vs. <30 %)

^d^HR value was extracted from subgroup analysis data of individual trial

^e^CTx includes low dose cytarabine and intensive chemotherapy

*Statistically significant

### Bias analysis

All five trials were open-labeled RCT. Random sequence generation and allocation concealment were performed adequately in all studies. The adequacy of blinding was judged by whether treatment response was evaluated by a third person who did not know the treatment group of the patients. Only one study [34] performed blinded assessment. Treatment response was assessed by unblinded reviewers in one study [36], and blinding status was unclear in three studies [33, 35, 37]. The baseline demographic characteristics were balanced in all trials (Table 1 and Additional file 1: Table S2), and potential sources of bias are described in Additional file 1: Table S3. OS and treatment response analyses showed significant heterogeneity, largely attributable to the HMA agent. The observed funnel plot asymmetry can be explained as a function of experimental agents (Additional file 1: Figure S1).

## Discussion

We performed a systematic review and pooled analysis to compare the outcomes of HMA vs. CCR in patients with AML and MDS. The combined analyses revealed statistically significant OS and CR/PR benefit with HMA therapy in comparison to CCR (Fig. 2). These results confirm that HMA are reasonable therapeutic options with survival advantage, especially for elderly and transplant ineligible AML and MDS patients.

Aberrant DNA methylation has been suggested as a dominant mechanism of MDS progression to AML, and patients with MDS and AML have been shown to have unique patterns and abundance of aberrant DNA methylation compared to normal controls [40, 41], thus representing a suitable therapeutic target. Azacitidine (5-azacytidine) is metabolized into decitabine (5-aza-2′-deoxycytidine), which forms a covalent protein-DNA adduct, depleting intracellular methyl-transferase, leading to reversal of DNA hypermethylation on tumor suppressor genes and induction of apoptosis [8, 10–13]. As such, DNA demethylation has been widely accepted as the primary mechanism of cytotoxicity of HMA and a previous study showed the association of *CDKN2B* (that encodes *p15*^*INK4B*^) pre-treatment methylation level with treatment response to azacitidine [42]. However, interestingly, our subgroup analyses demonstrated an association of OS benefit with azacitidine treatment, but not with decitabine (Table 2), similar to a recent retrospective study with AML patients that showed superior outcomes with azacitidine therapy in comparison to decitabine [25]. Furthermore, Fandy et al. showed that reversal of methylation on four tumor suppressor genes (*p15*^*INK4B*^, *CDH-1*, *DAPK-1*, and *SOCS-1*) had no prognostic impact on clinical response to the combination treatment of azacitidine and entinostat (histone deacetylase inhibitor) [43, 44]. Collectively, these results indicate potential cytotoxic mechanisms that are independent to DNA demethylation.

Both azacitidine and decitabine have been shown to induce DNA damage and cell cycle arrest, however, to different extents [45, 46], suggesting DNA damage as a possible underlying mechanism of HMA-induced cytotoxicity. However, a similar degree of γ-H2AX expression was observed in both responders and non-responders to azacitidine and entinostat treatment [43], questioning the role of DNA damage. Further studies are needed to define the role of DNA damage in the cytotoxic effect of azacitidine. In a recent study, azacitidine, but not decitabine, was shown to inhibit RNA methylation on cytosine 38 and 48 of tRNA^Asp^, which are target sites of DNMT2, and reduce the metabolic activity in myeloid cell lines. This suggests that azacitidine induces cytotoxicity via tRNA demethylation rather than DNA although the detailed mechanisms still remain to be answered. Also, Roulois *et al*. showed that the anti-tumor effect of low-dose decitabine may depend on viral mimicry, activating MDA5/MAVS/IRF7 RNA recognition pathway in colorectal cancer-initiating cells [47], and its role in myeloid neoplasms need further investigation.

Cytogenetic risk and BM blast counts are known to be independent prognostic factors in AML and MDS [39, 48–50]. However, in our study, subgroup analysis according to cytogenetic risk or BM blast count failed to show any RR difference (Table 2). Ninety-eight percent of MDS patients enrolled in three trials [33, 35, 36] had intermediate or high IPSS risk and two studies [34, 37] included only AML patients whose prognosis is known to be dismal, partially explaining why there was no subgroup difference.

Previously, LDAC has been shown to be associated with higher response rates compared to supportive care in elderly AML or MDS patients who were not candidates for allogeneic SCT [51]. As such, three trials [34, 36, 37] in the current analysis incorporated LDAC as their control regimens. In a subgroup analysis comparing HMA and LDAC in AML patients, the OS rate in LDAC treatment group was significantly lower than that of azacitidine, but not decitabine, suggesting that azacitidine may be a better therapeutic option in this patient group (Additional file 1: Figure S4).

We recognize several limitations of the current analysis. First, there was significant heterogeneity in the OS analysis (*I*^2^ = 89 %, *p* < 0.00001). The primary source of heterogeneity was experimental agents as shown in intra-subgroup homogeneity of three azacitidine trials [35–37] (*I*^2^ = 0 %, *p* = 0.38) as well as two decitabine trials [33, 34] (*I*^2^ = 0 %, *p* = 0.85). Although the proportion of females in decitabine studies was relatively higher, other demographic profiles or clinical parameters were not substantially different from azacitidine trials (Table 1 and Additional file 1: Table S2). BM morphologic abnormalities were shown to be associated with worse prognosis in *de novo* AML [52], and 32.4 % of patients (*n* = 158) in one azacitidine study [37] had AML-MRC, which may result in heterogeneity of treatment outcomes. However, a recent *ad hoc* study with AML-MRC patients [53] showed significantly higher OS and CR/CRi rates with azacitidine, similar to the original data, rendering this possibility less likely. Second, in one study [35], 53 % of patients who were initially randomized to the CCR group crossed over to azacitidine treatment. However, the outcomes of these patients were analyzed along with the CCR group, which may underestimate the efficacy of azacitidine. Third, inclusion of LDAC and IC as BSC in three studies [34, 36, 37] may have contributed to the heterogeneity, although the result from direct comparison of HMA and LDAC was not significantly different from the original analysis (Additional file 1: Figure S4). Fourth, schedule and administration method and number of cycles of HMA used in the individual studies were different, potentially generating additional heterogeneity based on previous studies that showed better treatment response with prolonged azacitidine treatment [54] and lower bioavailability of subcutaneous azacitidine compared to intravenous administration (AUC values 89 %) [55]. Lastly, the maximum tolerated dose (MTD) of decitabine is known to be 1500–2000 mg/m^2^/course [56]. However, decitabine trials in our analysis used a 10–25 mg/m^2^/day dose, which is significantly lower than the MTD. This is based on previous phase I trial that focused on pharmacodynamics rather than MTD [57]. The optimal dose-schedule of decitabine, an S-phase specific agent, needs be further investigated in the future study.

Our systematic review and pooled analyses have identified several areas that require further study. First, the mechanisms of action of HMA and their therapeutic targets remain to be poorly defined. Inhibition of tRNA has been suggested as a potential mechanism of azacitidine; however, details supporting this need further elucidation. Recently, activation of the MDA5/MAVS RNA recognition pathway was suggested as an underlying mechanism of decitabine-induced cytotoxicity in colorectal cancer-initiating cells, and its role in myeloid neoplasm requires further elucidation. Second, the benefit of initial treatment response with decitabine failed to translate into OS improvement, supporting the possibility that the superior OS with azacitidine may result from better disease control, the mechanisms of which remain unknown. In the same context, the optimal dose of decitabine still remains to be defined. Third, potential biomarkers that might have prognostic relevance with regards to response to HMA therapy, including nucleoside transporters like hCNT1 [58] and cytosine deaminase activity [59], also need further study. Finally, a second-generation HMA has been developed to reduce elimination of decitabine by cytidine deaminase, thereby increasing the in vivo exposure of decitabine. A recent phase I clinical trial with SGI-110 (dinucleotide of decitabine and deoxyguanosine) demonstrated a comparable safety profile to decitabine with a significantly longer half-life [60]. An ongoing phase II clinical trial (NCT01261312) will hopefully provide more data in regards to the clinical activity of second generation HMA.

## Conclusions

In an analysis of prospective randomized controlled trials in elderly patients with AML or MDS, HMA therapy was associated with improved response rates and OS in comparison to CCR, which included BSC, LDAC, and IC. Further analysis demonstrated that the observed survival benefit was restricted to azacitidine therapy, suggesting that azacitidine may be a better therapeutic option in AML and MDS patients. Finally, we also conclude on the need for additional mechanistic and epigenetic work to better understand mechanisms of action of HMA, optimal dosing strategies, and the identification of prognostic markers and biomarkers to help better predict and monitor response to therapy with these agents.

## Additional file

Additional file 1: Figure S1. Funnel plot. Figure S2. Subgroup analysis of OS rate from available data. Figure S3. Subgroup analysis of ORR from available data. Figure S4. Comparison of OS rates between HMA vs. LDAC in AML patients. Table S1. Search detail in PubMed, EMBASE, and Cochrane database of systematic review. Table S2. Additional characteristics of randomized trials. Table S3. Risk of bias assessment of studies according to Cochrane risk bias assessment tool. Table S4. Characteristics of trials (ad hoc studies) comparing HMAs and LDAC in AML patients. (DOC 1145 kb)
