# Supplementary material for: Targeting epigenetic pathways in acute myeloid leukemia and myelodysplastic syndrome: a systematic review of hypomethylating agents trials
Source: Clin Epigenetics. 2016 Jun 14;8:68. doi: 10.1186/s13148-016-0233-2 (PMC4908810; doi:10.1186/s13148-016-0233-2)
Supplement: Additional file 1: Figure S1. — Funnel plot. Figure S2. Subgroup analysis of OS rate from available data. Figure S3. Subgroup analysis of ORR from available data. Figure S4. Comparison of OS rates between HMA vs. LDAC in AML patients. Table S1. Search detail in PubMed, EMBASE, and Cochrane database of systematic review. Table S2. Additional characteristics of randomized trials. Table S3. Risk of bias assessment of studies according to Cochrane risk bias assessment tool. Table S4. Characteristics of trials (ad hoc studies) comparing HMAs and LDAC in AML patients. (DOC 1145 kb) [file 13148_2016_233_MOESM1_ESM.doc]

**Supplementary Materials to**

**Targeting Epigenetic Pathways in Acute Myeloid Leukemia and**

**Myelodysplastic Syndrome: A Systematic Review of**

**Hypomethylating Agents Trials**

Seongseok Yun MD, PhD, Nicole D. Vincelette PhD, Ivo Abraham PhD, Keith D. Robertson, Martin E. Fernandez-Zapico MD, Mrinal M. Patnaik MBBS

**Supplemental figure 1: Funnel Plot**

**
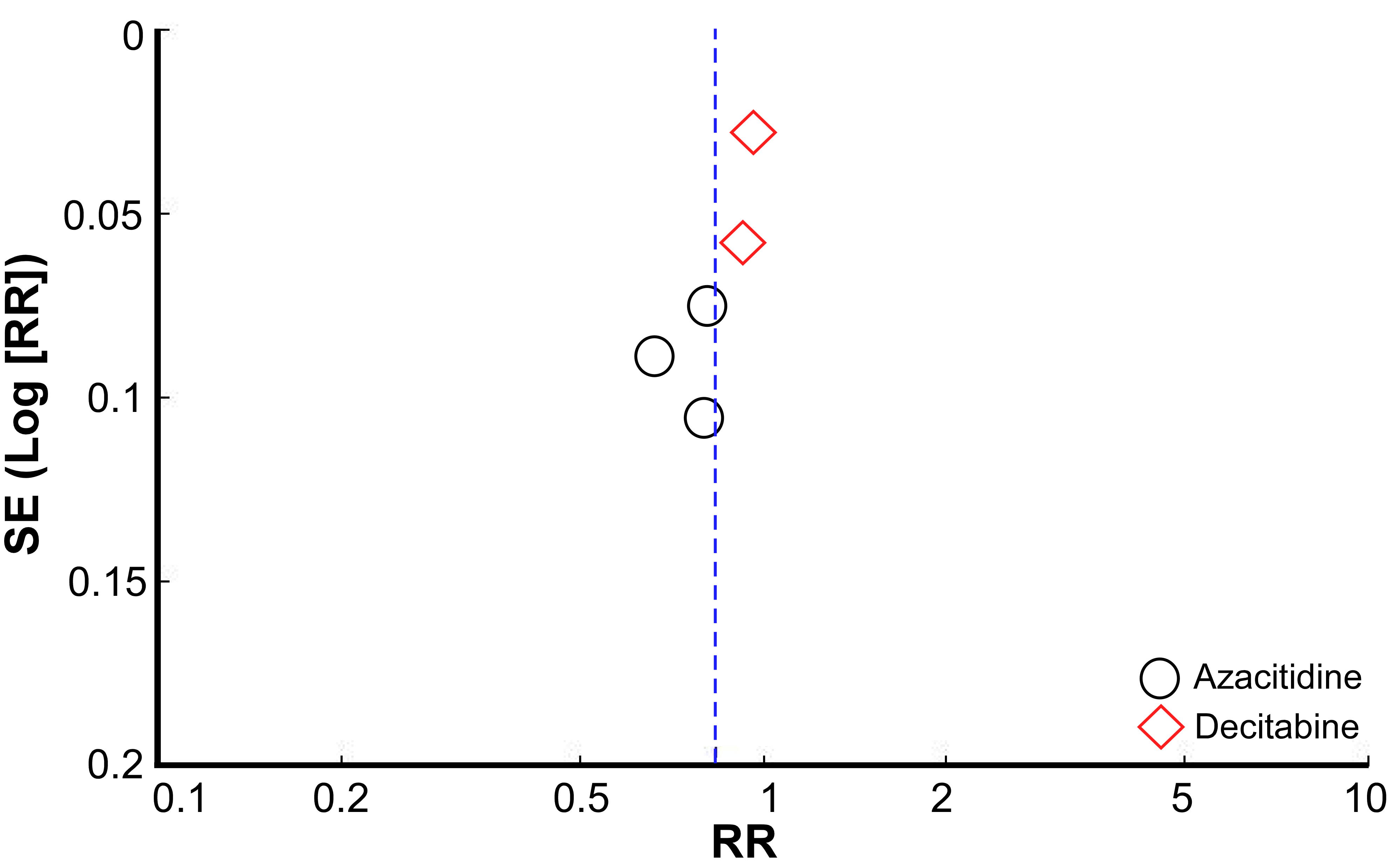
**

Funnel plot to evaluate the evidence of publication bias. Circle and diamond stand for azacitidine and decitabine, respectively.

**Supplementary Figure 2: Subgroup Analysis of OS rate from Available Data**

**S2A: Azacitidine vs. Decitabine**

**
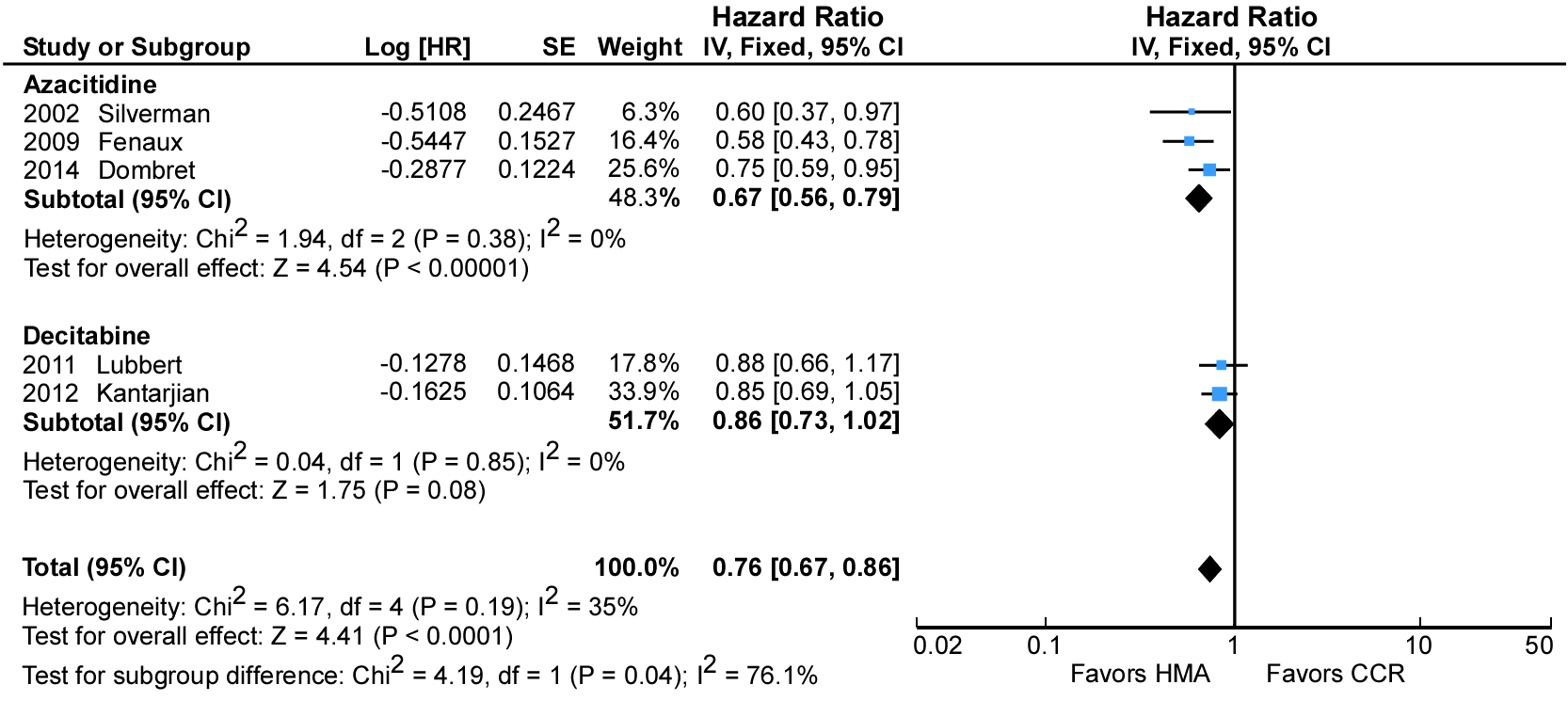
**

**S2B: Poor vs. Intermediate vs. good cytogenetic risk**

**
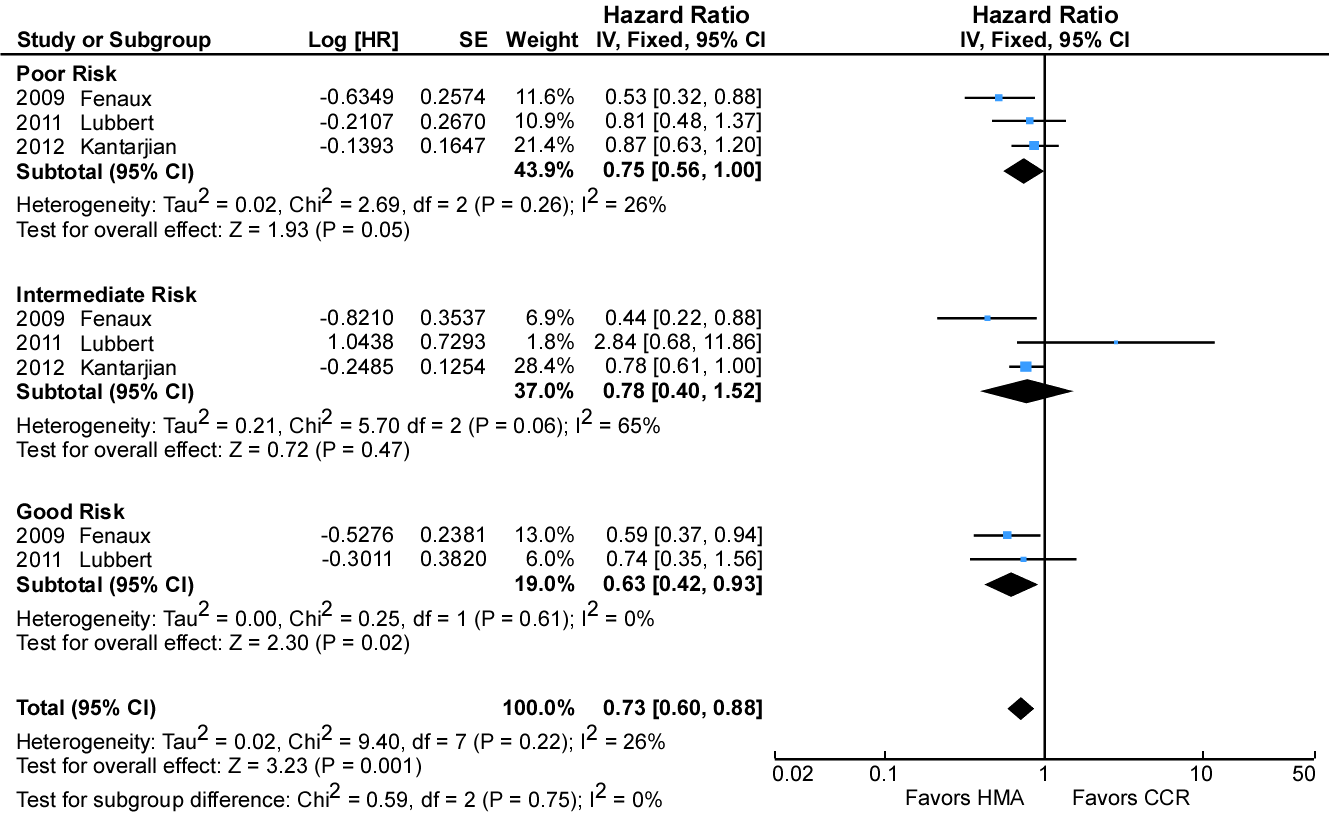
**

**S2C: BM Blast Count at least vs. less than 30%**

**
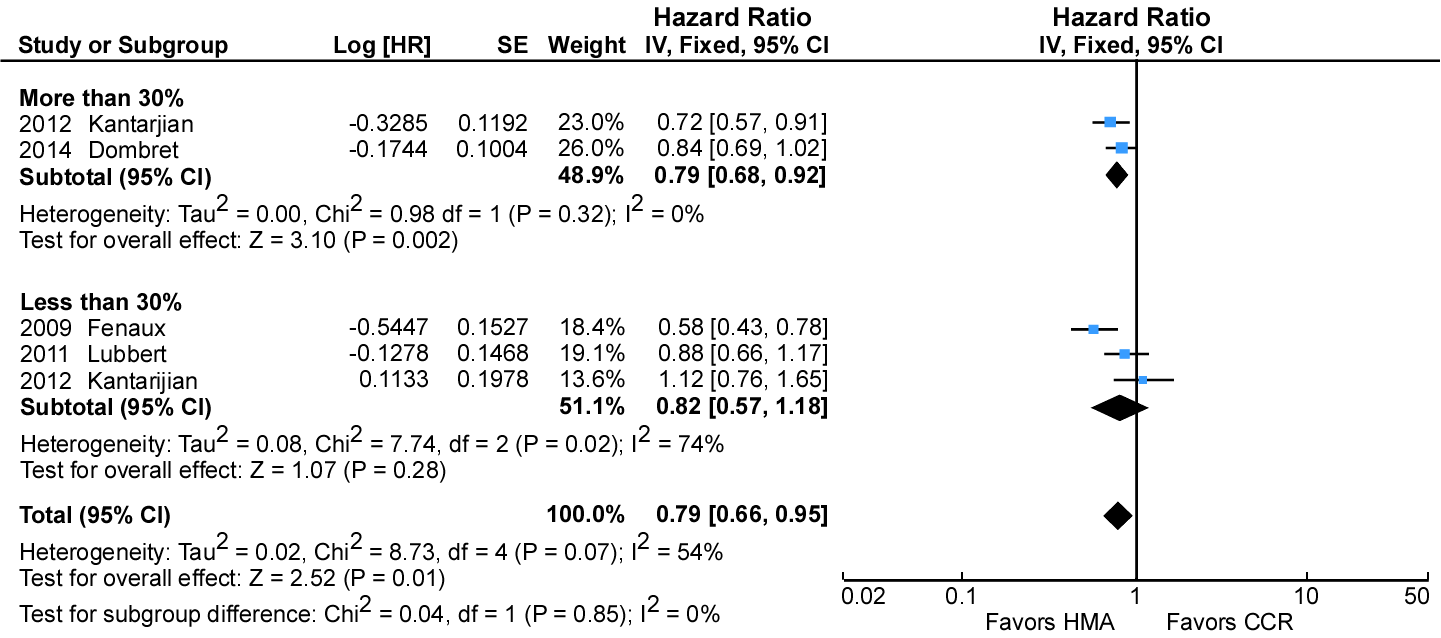
**

**S2D: BSC only vs. BSC and Chemotherapy**

**
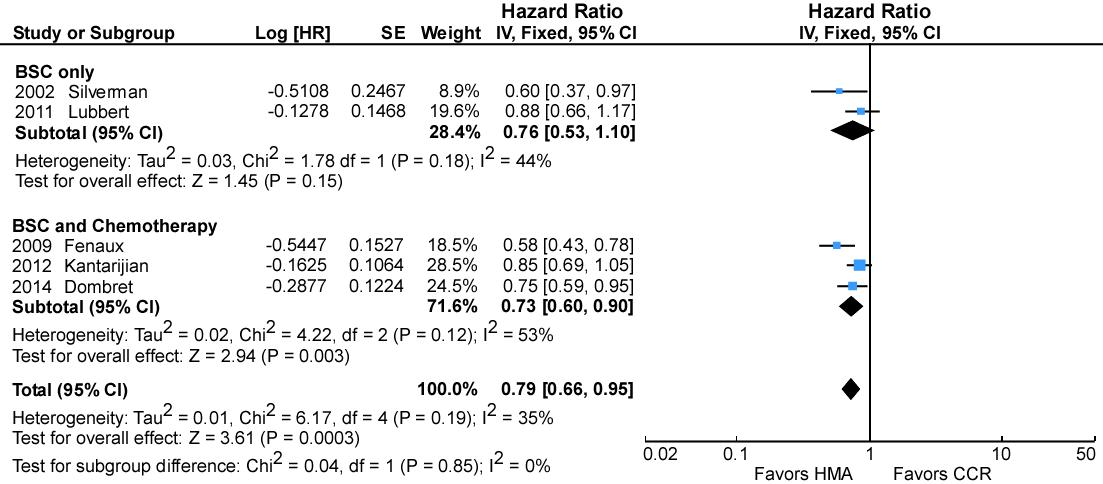
**

Forest plots of hazard ratios for OS from all available data. The size of the data markers (square) corresponds to the weight of the study in the analysis. The effects of interventions are calculated with the random effects model. Criteria used for subgroup analysis include experimental agents (azacitidine vs. decitabine) (A), cytogenetic risk group (poor vs. intermediate vs. good) (B), BM blasts count (≥30% vs. <30%) (C), and conventional care regimens (BSC only vs. BSC and chemotherapy) (D).

**Supplementary Figure 3: Subgroup Analysis of ORR from Available Data**

**
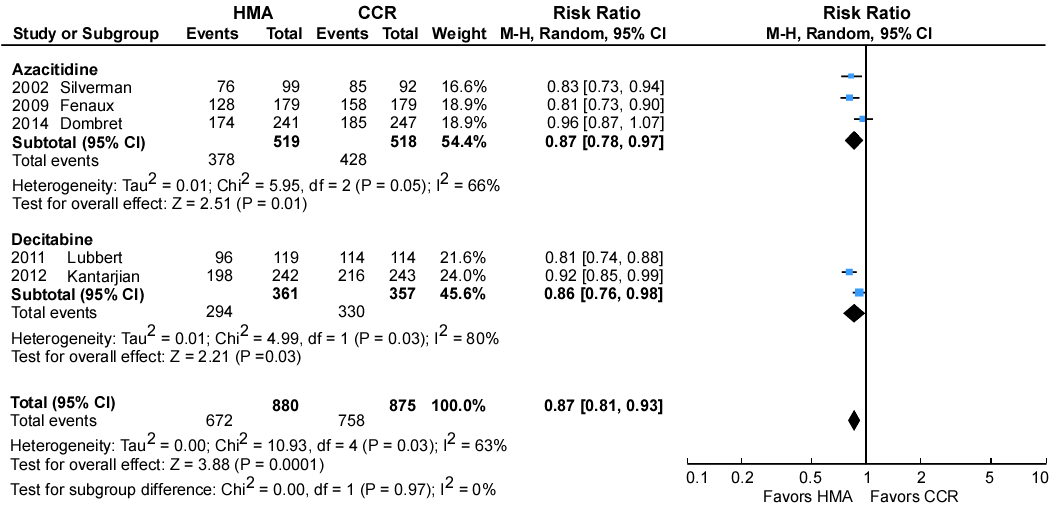
**

Forest plots of risk ratios for ORR (defined as rate of complete remission and partial remission) from all available data. Criteria used for subgroup analysis include experimental agents (azacitidine vs. decitabine). The size of the data markers (square) corresponds to the weight of the study in the analysis. The effects of interventions are calculated with the random effects model.

**Supplementary Figure 4: Comparison of OS rates between HMA vs. LDAC in AML patients.**

**
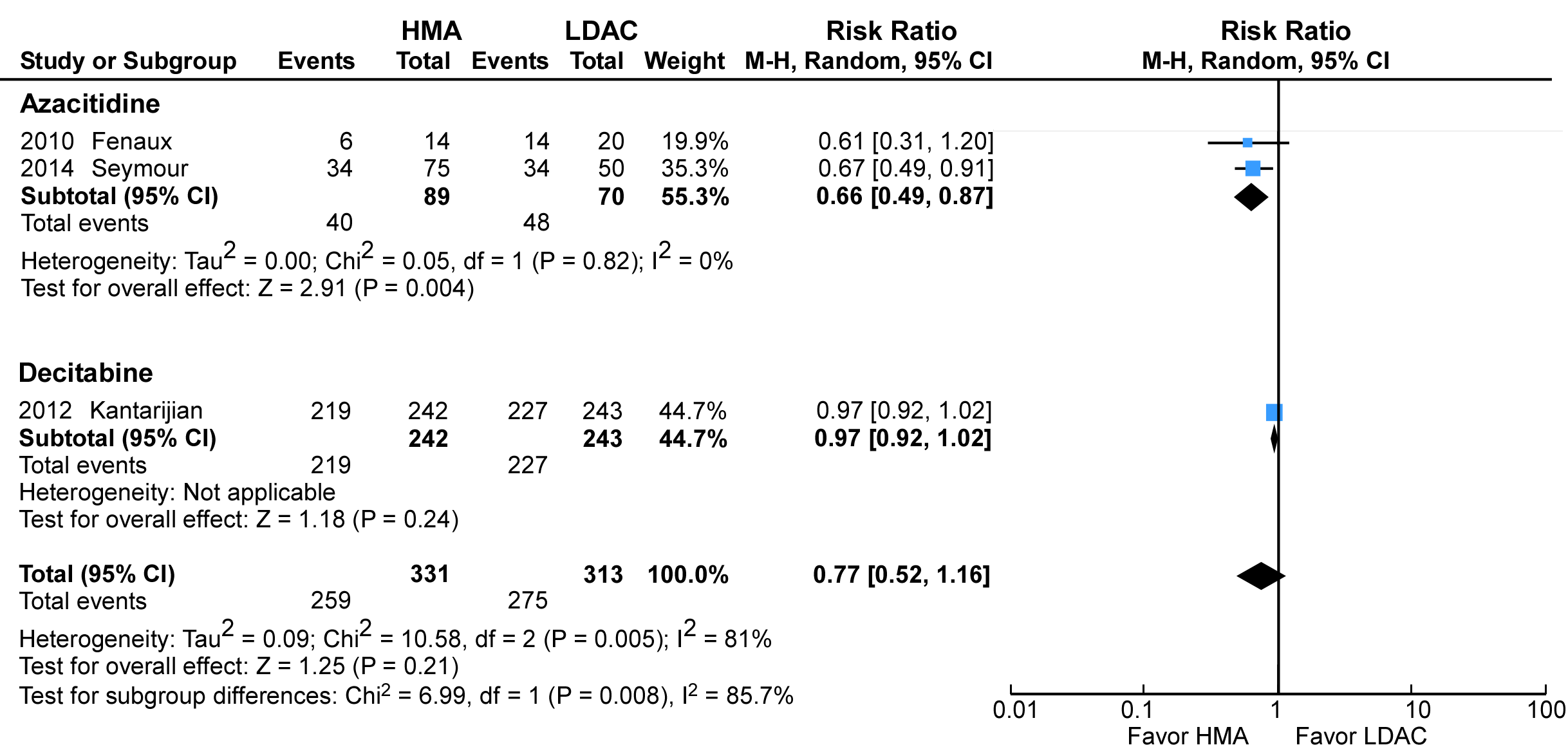
**

Forest plots of risk ratios for overall survival from *ad hoc* studies comparing HMAs and LDAC in AML patients. The size of the data markers (square) corresponds to the weight of the study in the analysis. The effects of interventions are calculated with the random effects model based on heterogeneity.

**Supplementary Table 1: Search Detail in PubMed, EMBASE and Cochrane Database of Systematic Review**

| **Study** | **Search strategies** | **No. of Results** |
| --- | --- | --- |
| **PubMed** | (“Myelodysplastic syndrome” [All Fields] OR “Acute Myeloid Leukemia” [All Fields]) AND (“Azacitidine” [All Fields] OR “5-azacytidine” [All Fields] OR “Decitabine” [All Fields] OR “5-aza-2’-deoxycytidine” [All Fields] OR “DNA methyltransferase” [All Fields]) AND (“controlled” [All Fields] OR “trial” [All Fields] OR “randomized” [All Fields]) AND ((“0001/01/01”[PDAT]: “2015/10/01”[PDAT]) AND “humans” [MeSH]) | 188 |
| **EMBASE** | 1. ‘Myelodysplastic Syndrome’ OR ‘Acute Myeloid Leukemia’  2. AND (‘Azacitidine’ OR ‘5-azacytidine’ OR ‘Decitabine’ OR ‘5-aza-2’-deoxycytidine’ OR ‘DNA methyltransferase’)/de  3. AND (‘Randomized Trial’)  3. AND (‘Human’)/de | 1. 68183  2. 3584  3. 265  4. 249 |
| **Cochrane Database of Systematic Review** | 1. ‘Myelodysplastic Syndrome’ OR ‘Acute Myeloid Leukemia’  2. ‘Azacitidine’ OR ‘Decitabine’ | 1. 8744  2. 53 |

The initial literature search was completed on 7/2014 and updated on 10/2015.

**Supplementary Table 2: Additional Characteristics of Randomized Trials**

| **Study** | **Study period^a^** | **Study design** | **Female (%)** | **ECOG (%)** | **Cytogenetic risk** | **IPSS risk stratification** | **Supportive Care** | **Cross-over (%)^b^** | **Tx response criteria** | **Median cycle of treatment (range)** |
| --- | --- | --- | --- | --- | --- | --- | --- | --- | --- | --- |
| **Silverman**  **2002^1,2^** | 1994-1996 | Open label  Multicenter  Phase 3 | 59 (31) | 0-1: 134 (70)  2: 14 (7)  3: 1 (0.5) | Complete cytogenetic data were only available for 81 patients | Low: 7 (9)  Intermediate: 59 (72)  High: 15 (19) | Transfusions  Antibiotics  No hematopoietic growth factors | 49 (53) | Used own criteria^1,2^ | NR |
| **Fenaux**  **2009^3,4^** | 2004-2006 | Open label  International  Multicenter  Phase 3 | 107 (30) | 0-1: 330 (92)  2: 23 (6) | Good: 167 (47)  Intermediate: 76 (21)  Poor: 100 (28)  Missing: 15 (4) | Intermediate: 164 (46)  High: 167 (47) | Transfusions  No hematopoietic growth factors | No crossover | Modified IWG 2003 Criteria^d^ | Azacitidine: 9 (4-15)  LDAC: 4.5 (2-8)  IC: 1 (1-3) |
| **Lubbert**  **2011^5,6^** | 2002-2007 | Open label  International  Multicenter  Phase 3 | 84 (36) | 0-1: 202 (87)  2: 31 (13) | Good: 67 (29)  Intermediate: 26 (11)  Poor: 108 (46)  Failure: 29 (12)  N/A: 3 (1) | Intermediate: 143 (61)  High: 88 (38)  Missing: 2 (1) | NR^c^ | No crossover | IWG 2000 Criteria^e^ | Decitabine: 4 |
| **Kantarjian**  **2012^7^** | 2006-2009 | Open label  International  Multicenter  Phase 3 | 197 (41) | 0-1: 367 (76)  2: 118 (24) | Intermediate: 306 (52)  Poor: 174 (36) ^f^ | NR | Transfusion  Erythropoietin | No crossover | Modified IWG 2003 Criteria^d^ | Decitabine: 4 (1-29)  LDAC: 2 (1-30) |
| **Dombret**  **2014^8,9^** | 2010-2014 | Open label  Multicenter  Phase 3 | NR | 0-2: 488 (100) | Intermediate/poor: 488 (100) ^g^ | NR | Transfusion | No crossover | Modified IWG 2003 Criteria^d^ | Azacitidine: 6 (1-28)  LDAC: 4 (1-25)  IC: 2 (1-3) |

^a^ The duration of patient enrollment.

^b^ Number of patients who received trial’s experimental drug in the subsequent treatment.

^c^ No information about best supportive care was reported.

^d^ Modified IWG 2003: Modified International Working Group Criteria 2003^10^.

^e^ IWG 2000: International Working Group Criteria 2000^11^.

^f^ SWOG categorization^12^.

^g^ There is no description about which cytogenetic risk stratification was used in this study.

Abbreviation: NR (not reported), LDAC (low dose cytarabine), IC (intensive chemotherapy).

**Supplementary Table 3: Risk of Bias Assessment of Studies According to Cochrane Risk Bias Assessment Tool**

| **Study** | **Random sequence generation** | **Allocation concealment** | **Blinding ^a^** | **Incomplete outcome data** | **Selective reporting** | **Other Source of Bias** |
| --- | --- | --- | --- | --- | --- | --- |
| **Silverman**  **2002 ^1,2^** | Adequate (minimization) | Adequate (central allocation) | Unclear | Adequate | Adequate | 1. 33% dose increase of experimental drug if no beneficial effect by day 57. 2. Total 49 supportive care patients were crossed over to azacitidine treatment. |
| **Fenaux**  **2009 ^3,4^** | Adequate (computer random number generation) | Adequate (central allocation) | Inadequate (unblinded review of efficacy analysis) | Adequate | Adequate | 1. The investigator preselection subgroups showed imbalances in patient demographics. |
| **Lubbert**  **2011 ^5,6^** | Adequate (minimization) | Adequate (central allocation) | Unclear | Adequate | Adequate | 1. Treatment cycle was extended up to 10 wks in case of insufficient regeneration of hematologic parameters to baseline values. |
| **Kantarjian**  **2012 ^7^** | Adequate (minimization) | Adequate (central allocation) | Adequate (blinded and central review of efficacy analysis) | Adequate | Adequate | 1. Patients indicated their preferred treatment choice either supportive care or LDAC based on physician’s advice. 2. Of patients in the decitabine and CCR groups, 1.7% and 5.8%, respectively received azacitidine treatment. 3. Of patients initially randomized to decitabine and supportive care, 8.7% and 4.5%, respectively received LDAC treatment. 4. Hydroxyurea was allowed until cycle 1 on day 15. 5. Study drug was not administered unless the absolute blasts count was less than 30,000 /µL. |
| **Dombret**  **2014 ^8,9^** | Unclear | Unclear | Unclear | Adequate | Adequate | 1. Data from abstract and presentation slide 2. 32.4% of patients had myelodysplastic related change (AML-MRC). |

^a^ The adequacy of blinding was judged by the blindness of outcome assessment since the overall survival is the primary outcome

**Supplementary Table 4: Characteristics of Trials (*ad hoc* studies) Comparing HMAs and LDAC in AML patients**

| **Study** | **Experimental Drugs** | **Median Age (Range)** | **Male**  **(%)** | ***de novo* AML (%)** | **Secondary AML ^†^ (%)** | **AML-MRC (%)** | **BM Blast (%)** | **ECOG PS (%)** | **Cytogenetic risk group** | **No. of patients** | | **Median F/U months (range)** |
| --- | --- | --- | --- | --- | --- | --- | --- | --- | --- | --- | --- | --- |
|  |  |  |  |  |  |  |  |  |  | **HMA** | **LDAC** |  |
| **Fenaux**  **2010^4^** | Azacitidine vs. LDAC | 70  (55-83) | 28 (82) | 0 (0) | 34 (100) | NR | < 30%: 34 (97)  ≥ 30%: 1 (3) | 0-1: 33 (97)  2: 0 (0)  Missing: 1 (3) | Favorable: 0 (0) ^¶^  Intermediate: 27 (79)  Unfavorable: 6 (18)  Missing: 1 (3) | 14 | 20 | 20.1 (0.03-38.4) |
| **Kantarjian**  **2012^7^** | Decitabine vs. LDAC | 73  (64-91) | 288 (60) | 312 (64) | 171 (36) | NR | < 30%: 123 (25)  ≥ 30%: 347 (72) | 0-1: 367 (76)  2: 118 (24) | Intermediate: 306 (52) ^δ^  Poor: 174 (36) | 242 | 243 ^‡^ | NR ^Ω^ |
| **Seymour**  **2014^13^** | Azacitidine vs. LDAC | 75  (65-87) | NR | 65 (41) | 93 (59) | 158 (100) ^Ф^ | < 30%: 0 (0)  ≥ 30%: 158 (100) | 0-1: 119 (75) ^Ф^  2: 39 (25) | Intermediate: 54 (43) ^θ^  Poor: 71 (57) | 75 | 50 | NR |

^†^ Definition of *de novo* and secondary AML followed the revised recommendations of the International Working Group^10^.

^‡^ 28 (12%) patients were initially randomized to best supportive care, however 4.5% of these patients eventually received LDAC treatment.

^¶^ Classified patient into favorable (inv(16), t(8,21)), unfavorable (-7/7q- or complex), or intermediate (all others including normal) karyotypes.

^Ф^ Total number of patient in the original study was 158 (azacitidine (n=75), LDAC (n=50), IC (n=13), BSC (n=20)).

^Ω^ Patients were followed until death or loss to follow-up.

^δ^ Cytogenetics risk stratification was performed by SWOG categorization^12^.

^θ^ Information about cytogenetics risk stratification criteria is missing.

Abbreviation: AML (acute myeloid leukemia), HMA (DNA hypomethylating agents), NR (not reported), LDAC (low dose cytarabine), MRC (myelodysplastic related change).
